# A case report of progressive obstruction of Ex-PRESS miniature glaucoma shunt after transient flat anterior chamber and treatment using Nd:YAG laser

**DOI:** 10.1186/1471-2415-15-2

**Published:** 2015-01-08

**Authors:** Masaki Tanito, Ichiya Sano, Akihiro Ohira

**Affiliations:** Division of Ophthalmology, Matsue Red Cross Hospital, 200 Horo-machi, Matsue, Shimane, 690-8506 Japan; Department of Ophthalmology, Shimane University Faculty of Medicine, Izumo, Japan

**Keywords:** Ex-PRESS miniature glaucoma shunt, Trabeculectomy, Surgical complication, Flat anterior chamber, Hypotony, Neodymium:yttrium-aluminium-garnet (Nd:YAG) laser

## Abstract

**Background:**

We report a case of Ex-PRESS miniature glaucoma shunt obstruction resulting from progressive iris synechial formation after transient anterior chamber shallowing.

**Case presentation:**

A 68-year-old woman with pseudoexfoliation glaucoma in her right eye underwent filtration surgery with implantation of the Ex-PPESS shunt (model P-50, Alcon Japan, Tokyo, Japan) in combination with intra-surgical 0.04% mitomycin C use. After the anterior chamber injection of viscoelastic material and 100% sulfur hexafluoride gas for treatment of early postoperative over filtration, the intraocular pressure (IOP) was controlled between 9 and 12 mmHg. On postoperative day 121, gonioscopy showed that synechial formation around the shunt obstructed the axial port leaving the relief port opened. On postoperative day 274, the intraocular pressure increased to 40 mmHg and synechiae obstructed both the axial and relief ports. Dispersion of iris tissue by neodymium:yttrium-aluminium-garnet (Nd:YAG) laser (2 mJ, one shot to each port) opened both ports and immediately lowered the IOP, leaving peripheral anterior synechiae around the shunt. Up to postoperative day 400, the IOP was controlled between 13 and 15 mmHg, and the cystic bleb was maintained.

**Conclusion:**

The synechiae formed gradually extends around the shunt’s shaft and can result in later external obstruction of the relief port. The current case requires further follow-up since synechiae remaining around the shaft can cause future obstruction. We emphasize the fact that, if the iris synechiae to the shunt once formed, it can progress and obstruct the shunt ports later.

## Background

Trabeculectomy with the Ex-PRESS miniature glaucoma shunt under the scleral flap has intraocular pressure (IOP)-lowering effects and a safety profile similar to standard trabeculectomy [1, 2] but may be associated with a lower rate of early postoperative complications [3, 4]. We report a case of Ex-PRESS shunt obstruction resulting from progressive iris synechial formation after transient anterior chamber (AC) shallowing. This case was treated successfully using neodymium:yttrium-aluminium-garnet (Nd:YAG) laser.

## Case presentation

**Figure 1:**
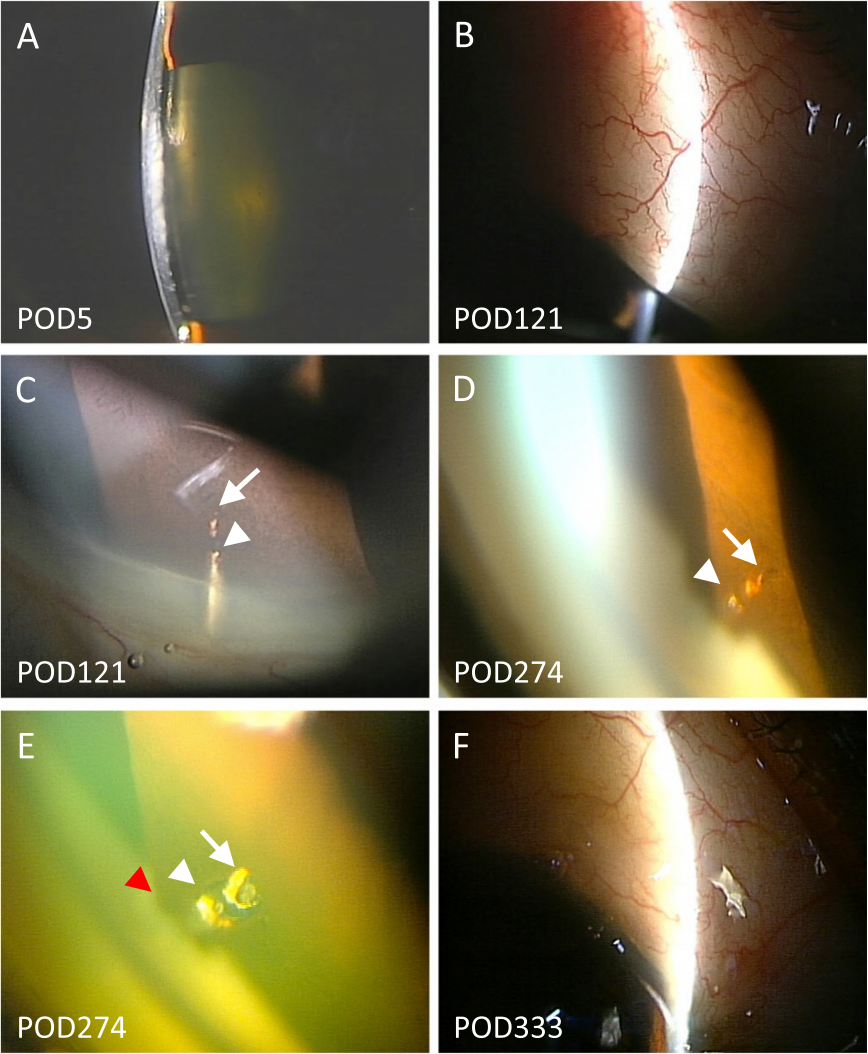
**Case.** A flat anterior chamber is observed on postoperative day (POD) 1 **(A)**. On POD 121, a well-formed filtration bleb is seen **(B)**; synechial formation to the shunt obstructs the axial port **(C**, arrow) but not the relief port **(C**, arrowhead). On POD 274, synechiae obstruct both the axial **(D**, arrow) and relief ports **(D**, arrowhead). The obstruction opens after neodymium:yttrium-aluminium-garnet laser therapy (E, arrow and arrowhead), leaving synechiae around the shunt **(E**, red arrowhead). The cystic bleb is reformed and maintained up to POD 333 **(F)**.

A 68-year-old woman with pseudoexfoliation glaucoma in her right eye underwent filtration surgery with implantation of the Ex-PPESS shunt (model P-50, Alcon Japan, Tokyo, Japan). Preoperatively, the IOP was 25 mmHg with use of four ocular hypotensive medications, and the AC angle was open (Shaffer grade 3). The shunt was inserted into the AC through a scleral tunnel under a half-thickness 4×4-mm scleral flap followed by closure with three interrupted 10–0 nylon sutures. The scleral tunnel was created by an AC paracentesis with a 25-gauge needle at the anterior edge of the scleral spur that ran parallel to the iris plane. Before shunt insertion, 0.04% mitomycin C was applied for 3 minutes followed by rinsing with 100 ml of balanced salt solution. On postoperative day (POD) 1, the IOP was 5 mmHg, and slight shallowing of the AC and a choroidal detachment (CD) were observed. On POD 5, the IOP was 5 mmHg, the AC flattened (Figure 1A), and the CD worsened. On the same day, viscoelastic material was injected to reform the AC. On POD 8, the IOP was 5 mmHg, and the flat AC and CD remained. On the same day, viscoelastic material and 100% sulfur hexafluoride gas were injected into the AC. As a result, the AC deepened and the CD subsided, leaving the axial port obstructed by the iris. After then, the IOP was controlled between 9 and 12 mmHg. On POD 121, a cystic bleb was seen (Figure 1B); gonioscopy showed that synechial formation around the shunt obstructed the axial port (Figure 1C, arrow) leaving the relief port opened (Figure 1C, arrowhead). On POD 274, the IOP increased to 40 mmHg and the bleb flattened; synechiae obstructed both the axial (Figure 1D, arrow) and relief ports (Figure 1D, arrowhead). Dispersion of iris tissue by Nd:YAG laser (2 mJ, one shot to each port) opened both ports (Figure 1E, arrow and arrowhead) and immediately lowered the IOP, leaving peripheral anterior synechiae around the shunt (Figure 1E, red arrowhead). Up to POD 400, the IOP was controlled between 13 and 15 mmHg, and the cystic bleb was maintained (Figure 1F).

## Conclusions

In a case series of 345 Ex-PRESS shunt implantations, Kanner et al. [5] reported that the most common device-related complication was shunt obstruction (6 eyes, 1.6%). Of the six shunts, one was blocked with vitreous, but no visible obstruction was observed in the others. In those five shunts, Nd:YAG laser treatment of the shunt tip resulted in dispersion of whitish particles near the tube tip, bleb elevation, and IOP reduction. Bagnis et al. [6] reported a case of obstruction in which the axial port was plugged with iris strands; the obstruction was treated with Nd:YAG laser. We confirmed that transient shallowing of the AC can cause synechiae to develop to the shunt and obstruct the axial port. The synechiae formed gradually and extended around the shaft and can result in later external obstruction of the relief port. The current case required further follow-up since synechiae remaining around the shaft can cause future obstruction.

With this case report, we emphasize the fact that, if the iris synechiae to the shunt once formed, it can progress and obstruct the shunt ports later.

## Consent

Written informed consent was obtained from the patient for publication of this Case report and any accompanying images. A copy of the written consent is available for review by the Editor of this journal.

## Abbreviations

AC: Anterior chamber
CD: Choroidal detachment
IOP: Intraocular pressure
Nd: YAG: Neodymium:yttrium-aluminium-garnet
POD: Postoperative day.
